# Proposing a “Brain Health Checkup (BHC)” as a Global Potential “Standard of Care” to Overcome Reward Dysregulation in Primary Care Medicine: Coupling Genetic Risk Testing and Induction of “Dopamine Homeostasis”

**DOI:** 10.3390/ijerph19095480

**Published:** 2022-04-30

**Authors:** Eric R. Braverman, Catherine A. Dennen, Mark S. Gold, Abdalla Bowirrat, Ashim Gupta, David Baron, A. Kenison Roy, David E. Smith, Jean Lud Cadet, Kenneth Blum

**Affiliations:** 1 The Kenneth Blum Institute on Behavior & Neurogenetics, Austin, TX 78701, USA; pathmedical@gmail.com (E.R.B.); catherine.a.dennen@gmail.com (C.A.D.); 2 Department of Psychiatry, Washington University School of Medicine, St. Louis, MO 63110, USA; drmarksgold@gmail.com; 3 Department of Psychiatry, Tulane School of Medicine, New Orleans, LA 70112, USA; kenroymd@gmail.com; 4 Department of Molecular Biology, Adelson School of Medicine, Ariel University, Ariel 40700, Israel; bowirrat@gmail.com; 5 Future Biologics, Lawrenceville, GA 30043, USA; ashim6786@gmail.com; 6 Division of Addiction Research & Education, Center for Psychiatry, Medicine & Primary Care (Office of Provost), Western University Health Sciences, Pomona, CA 91766, USA; dbaron@westernu.edu; 7 Department of Cellular and Molecular Pharmacology, University of California San Francisco, San Francisco, CA 94158, USA; dsmith4619@aol.com; 8 The Molecular Neuropsychiatry Research Branch, NIH National Institute on Drug Abuse, Baltimore, MD 21224, USA; jcadet@intra.nida.nih.gov

**Keywords:** Brain Health Check (BHC), memory, attention, neuropsychiatry, qEEG, P300, substance use disorder (SUD), reward dysregulation, genetic addiction risk scores, epigenetics

## Abstract

In 2021, over 100,000 people died prematurely from opioid overdoses. Neuropsychiatric and cognitive impairments are underreported comorbidities of reward dysregulation due to genetic antecedents and epigenetic insults. Recent genome-wide association studies involving millions of subjects revealed frequent comorbidity with substance use disorder (SUD) in a sizeable meta-analysis of depression. It found significant associations with the expression of NEGR1 in the hypothalamus and DRD2 in the nucleus accumbens, among others. However, despite the rise in SUD and neuropsychiatric illness, there are currently no standard objective brain assessments being performed on a routine basis. The rationale for encouraging a standard objective Brain Health Check (BHC) is to have extensive data available to treat clinical syndromes in psychiatric patients. The BHC would consist of a group of reliable, accurate, cost-effective, objective assessments involving the following domains: Memory, Attention, Neuropsychiatry, and Neurological Imaging. Utilizing primarily PUBMED, over 36 years of virtually all the computerized and written-based assessments of Memory, Attention, Psychiatric, and Neurological imaging were reviewed, and the following assessments are recommended for use in the BHC: Central Nervous System Vital Signs (Memory), Test of Variables of Attention (Attention), Millon Clinical Multiaxial Inventory III (Neuropsychiatric), and Quantitative Electroencephalogram/P300/Evoked Potential (Neurological Imaging). Finally, we suggest continuing research into incorporating a new standard BHC coupled with qEEG/P300/Evoked Potentials and genetically guided precision induction of “dopamine homeostasis” to diagnose and treat reward dysregulation to prevent the consequences of dopamine dysregulation from being epigenetically passed on to generations of our children.

## 1. SUD Global Pandemic

Addiction is a global pandemic that has negatively affected millions of people [1]. Although addiction itself is not a diagnostic term in the International Classification of Diseases 10th revision (ICD-10), it is still widely used by professionals and the public [1]. When it comes to diagnostic terminology, “addiction” was previously classified as substance abuse and substance dependence in the fourth edition of the Diagnostic and Statistical Manual of Mental Disorders (DSM) [2]. The DSM-5 consolidated the terminology into substance use disorder (SUD) with a severity scale [3]. However, federal agencies continue to use different/multiple terms such as SUD, alcohol use disorder (AUD), opioid use disorder (OUD), addiction, substance dependence, substance abuse, and experimental gateway, etc. [4,5,6,7,8]. For the purpose of this paper, SUD is all-encompassing. 

The National Center for Drug Abuse Statistics (NCDAS) reports that ~12% of Americans over the age of 12 use illicit drugs, and if alcohol and tobacco are included, then the percentage increases to ~60 [9]. In the United States (US), ~50% of teenagers have misused a drug at least once and ~61% of teenagers have abused alcohol by 12th grade [10,11]. In addition, 70% of users who experiment with illegal drugs before age 13 develop SUD within the next 7 years, compared to 27% of those who experiment with illegal drugs after age 17 [9]. Furthermore, many individuals with SUD also suffer from polysubstance abuse. For example, according to the Substance Abuse and Mental Health Services Administration (SAMHSA), ~35% of adults with illicit drug use disorder also have AUD [12]. Alcohol is one of the most commonly used drugs, and AUD is the most common type of SUD in the US [13,14]. The lifetime prevalence of AUD is ~29%, the prevalence of binge drinking in young adults (age 18–25) is ~31%, and ~70% of Americans report having alcohol in the past year [9,10,15]. SUD global severity, by country, is also underreported and inconsistent, ranging from 5 to 20%, depending on the agency [16,17,18,19,20,21,22].

SUD reporting is underestimated in the US due to stigma, confusion, lack of precision in reporting medical causes of death, poor self-reporting, and confusing/lack of uniform terminology, etc. [23,24,25,26,27,28,29,30,31,32,33,34,35]. Patients with SUD often have co-occurring neuropsychiatric/medical disorders, and thus, their deaths can be attributed to any one of their comorbidities, leading to death certificate discrepancies. Furthermore, SUD is often the tipping point to death in patients with medical comorbidities [36]. Drug overdose deaths are often reported as accidents (cars, machinery, military, prescription mistakes, etc.), heart attacks, sudden cardiac deaths, strokes, etc. [23,24,25,26]. In 2019, SUD was reported as the third and/or tenth leading cause of death classified as accidental or suicide, respectively [37]. Approximately 50% of all death certificates and 20–30% of drug overdose death certificates that the CDC receives are inaccurate or have incomplete causes of death [25,35]. These inaccuracies result in the underreporting of deaths attributed to SUD. SUD mortality will likely continue to increase until a more accurate and reliable system is implemented [35]. Finally, we believe that in those cases with the presence of DNA antecedent polymorphisms across the brain reward circuitry, it might suggest that SUD is not easily reversed and may indeed be a life-long condition if untreated. Nevertheless, our new knowledge related to the role of environment and epigenetic influence on gene expression that includes acetylation (positive expression) and methylation (negative expression), the presence of SUD in an individual could become a reversable phenotype if the epigenetic repair becomes a life-style change, including induction of brain circuitry homeostasis.

Therefore, we believe that the US medical system, especially pediatrics, must implement and utilize standard neuropsychiatric metrics and imaging in the diagnosis and treatment of SUD until the number of SUD deaths becomes zero.

## 2. Economic Burden of SUD Crisis

During the COVID-19 pandemic, reported opioid youth deaths increased by 25%, from ~75,000 to ~100,000, with an average age of 24, ~70% men [38,39,40,41]. SUD is estimated to cost the US ~1 trillion dollars, i.e., 700 billion National Institute on Drug Abuse (NIDA), 300 billion National Institute on Alcohol Abuse and Alcoholism (NIAAA), but this value is extremely underestimated [42,43,44,45,46,47,48,49,50]. If we include all SUD, + food, + neuropsychiatric/medical comorbidities, the annual cost might be as high as 4 trillion, including lost work productivity [36,42,43,44,45,46,47,48,49,50,51]. Furthermore, a recent study involving young individuals (age 10–24) showed that, over a 5-year period (2015–2019), ~1.25 million years of life were lost [52]. Even with current epidemiological data, the loss to the US is approximately 7 million dollars in life years (loss of approximately 50 years of a person’s life by dying at 24 of SUD) [45,47]. If an average person’s lifetime financial productivity is ~USD 4 million dollars, then the loss to the gross national product (GNP)/future economy is ~USD 30 trillion dollars over time [45,47]. The direct and indirect medical comorbidities of SUD are so vast due to reward deficiency syndrome that it may account for up to 75% of the total health expenditure, or ~USD 4 trillion dollars (Table 1) [36,42,43,44,45,46,47,48,49,50,51,53].

Secondary costs of SUD, especially food addiction/obesity, are in the trillions of dollars because of medical, neuropsychiatric, and social comorbidities (Table 1 and Table 2) [36,42,43,44,45,46,47,48,49,50,51,168]. Most SUD patients also concomitantly suffer from an abundant number of neuropsychiatric and medical findings, referred to by some as global dopamine dysfunction. In contrast, the premorbid neuropsychiatric medical findings that predict progression to SUD are less well known (Table 2). However, some common precursors to SUD include school failure and problems with family, memory, and attention [32,33,34] (Table 2).

## 3. Failure of the Current Paradigm

NIDA defines SUD as a chronic relapsing disorder. As a result, the long-term success rate of Alcoholics Anonymous (AA) and rehab groups is very low, except on the rare occasion when patients follow treatment guidelines [6,181,182]. According to NIDA, the relapse rate for SUD is 40–60% and “resembles those of other chronic diseases such as diabetes, hypertension, and asthma” [183]. Therefore, longitudinal studies must follow patients with SUD over the course of years (we recommend at least five) or even a lifetime for verified results [184,185]. Furthermore, a placebo response may last as long as a year with the buoyant attention of a healthcare practitioner [186].

Neuropsychiatric disorders are common in the US, and according to the National Institute of Mental Health, nearly one in five US adults has a mental illness [187]. In addition, ~45% of individuals with SUD have co-occurring neuropsychiatric disorders [12]. Neuropsychiatric disorders and SUD influence each other, and their combined presentation can result in more severe functional impairment, poorer treatment outcomes, higher treatment costs, and increased mortality and morbidity [188]. Many individuals with SUD and/or neuropsychiatric disorders do not receive treatment [187,188]. In a study by Han et al., only ~9% of adults with SUD and co-occurring neuropsychiatric disorders received both mental health care and substance use treatment, while ~52% received neither form of treatment [188]. Additionally, they reported that ~3–13% received only substance use treatment, and ~34–44% received only mental health care [188]. These findings reveal a significant disparity between the prevalence of SUD and co-occurring neuropsychiatric disorders prevalence and treatment rates.

Solving the SUD pandemic would resolve the US economic crisis, including debt service. One important dilemma related to the treatment of opioid dependence is the widespread use of opioids to specifically treat the unfortunate victims of OUD. While the prescribing of opioids (i.e., Buprenorphine) has reduced harm, it does not result in prophylaxis [189]. To achieve some reasonable solution in terms of treatment, relapse prevention, and prophylaxis, one novel therapeutic should include genetic risk assessment and induction of dopamine balance [190]. Many addiction programs are opting for the use of the non-addicting and relatively safe narcotic antagonist Naltrexone despite its associated poor compliance, even in the injectable form [191,192,193]. An analysis conducted at Stanford University suggested that the number of unwanted deaths from prescription opioids and street heroin will continue to increase if no changes are made to the currently available treatment, prevention, and public health approaches [194].

Studies have shown that Naltrexone is beneficial by attenuating cravings via “psychological extinction” and reducing relapse. In addition, research performed by Blum’s group has shown that Buprenorphine is the current medication-assisted treatment (MAT) of choice, but injectable Naltrexone plus an agent to enhance dopaminergic function and tone may rekindle interest amongst addiction physicians and patients [195]. Previously, an open-label investigation in humans showed improvement in Naltrexone compliance and outcomes with dopamine augmentation using the pro-dopamine regulator referred to as KB220 (262 days) compared to naltrexone alone (37 days) [195]. This well-studied complex consists of amino-acid neurotransmitter precursors and enkephalinase inhibitor therapy compared to standard treatment [196]. Consideration of this novel paradigm shift may assist in not only addressing the current opioid epidemic but also the broader issue of reward dysregulation.

It is important to recognize that SUD may also occur with certain palatable foods such as sugar and may also become highly addictive to the individual. It is well known that over 90 percent of people with obesity have an abnormal romance with carbohydrates. It is also well-known that there is strong evidence that common neurochemical mechanisms are observed in both animal and human imaging studies [48,180].

## 4. SUD—A Neurological Disorder

SUD is widely recognized as a neurological disorder. However, despite this, there are currently no standard, routine, objective brain assessments being performed, and without standardization, patients with neurological/brain disorders/dysfunction will not be able to receive the treatment they need. For example, it took approximately 40 years to obtain a core set of cardiac tests, e.g., electrocardiogram (EKG), echocardiogram, and blood tests, etc., which ultimately halted the bypass cardiac pandemic. Brain health needs to have a similar stepwise approach that parallels those used to treat cardiac disease because, without routine objective assessments, clinical studies lack reliability, accuracy, dependability, and reproducibility (Table 3). Therefore, utilizing the currently available inexpensive objective testing of premorbid memory, attention, and neuropsychiatry, with the possible addition of supplemental imaging and genotyping, would greatly improve brain/mind health and help combat the addiction pandemic (Table 3) [180,197].

## 5. Our Theoretical Construct for Primary Care and Reward Dysregulation

Based on the literature review, it is our opinion that a novel, cost-effective Brain Health Checkup that involves the domains: Memory, Attention, Neuropsychiatry, and Neurological Imaging may become the new standard of care in pediatric medicine, starting with children aged five and especially following Genetic Addiction Risk Score (GARS) testing (for curiosity or especially in a family tree robust with Reward Deficiency Syndrome (RDS) behaviors such as AUD, etc.). This checkup would be referred to as a “Brain Health Checkup” (BHC). Utilizing primarily PUBMED, the current paper herein, reviews over 36 years of virtually all the computerized and written-based assessments of Memory, Attention, Neuropsychiatry, and Neurological imaging. This research found the following tests to be the most beneficial for each of the aforementioned domains and recommends their use in the BHC: Central Nervous System Vital Signs (Memory), Test of Variables of Attention (Attention), Millon Clinical Multiaxial Inventory III (Neuropsychiatric), Quantitative Electroencephalogram/P300/Evoked Potential (Neurological Imaging). Obviously, these denoted domains should not be limited but in our opinion be included in any BHC to be effective in diagnoses in primary care and reward dysregulation.

## 6. SUD Induced Memory Loss

SUD is a total brain disorder that causes brain injury, delayed processing, decreased memory, attention abnormalities, neuropsychiatric disturbance, and MRI results that parallel dementia/mild cognitive impairment (MCI) (Table 4) [197,198,199,200,201,202,203,204,205,206,207,208,209,210,211,212,213,214,215,216,217,218,219,220,221,222,223,224,225,226]. Brain atrophy/damage is found in MCI, moderate cognitive impairment (MoCI), and dementia [197,198,199,200,201,202,203,204,205,206,207,208,209,210,211,212,213,214,215,216,217,218,219,220,221,222,223,224,225,226]. MRI results can be confusing if not performed serially, as the worsening of atrophy is more predictive of dementia. SUD may cause more injury to the dorsal lateral prefrontal cortex and mesial temporal lobe [198]. Additionally, studies have shown that SUD may cause slightly greater damage than MCI [197,198,199,200,201,202,203,204,205,206,207,208,209,210,211,212,213,214,215,216,217,218,219,220,221,222,223,224,225,226]. The mechanism of SUD brain atrophy is the same as dementia, called retrogenesis, which is the opposite of neurogenesis [198,227,228,229,230,231,232,233,234,235].

Out of all the memory tests reviewed, the Central Nervous System Vital Signs (CNSVS) assessment was found to be the best. The CNSVS is a computerized neuropsychological test used to evaluate the neurocognitive status of patients, which includes mental processes that range from simple motor performance, attention, and memory to executive functions [236,237]. It includes 10 normed objective neurocognitive tests: verbal memory, visual memory, finger tapping, symbol digit coding, shifting attention, continuous performance, perception of emotions, Stroop test, non-verbal reasoning, and four-part continuous performance [236,237]. CNSVS was found to be the best memory test because it is SOC2 certified, registered with the FDA, available globally, economical at ~USD 35, has greater than 99% compliance, and only takes ~30 min to complete [236,237,238,239,240]. CNSVS has high appeal because complex attention correlates with better SUD function and possible prevention [236,238,239,241,242]. CNSVS has no practice learning effect (test–retest probability) and becomes a reliable indicator of deterioration or improvement over time [236]. The presidential Montreal Cognitive Assessment (MoCA) test correlates well with CNSVS results [242].

## 7. SUD Induced Attention Issues

Attention deficits are common in SUD patients [243,244,245,246]. In fact, research has shown that of adults presenting with SUD, 20–30% have concurrent ADHD, and 20–40% of adults diagnosed with ADHD also have a history of SUD [180,243,244,245,246]. Therefore, the Test of Variables of Attention (TOVA), which is an attention screening test, would be useful in the diagnosis and subsequent treatment of SUD. The TOVA measures omission errors (inattention), commission errors (impulsivity), response time, response time variability (consistency), commission error response time, post-commission error response time, anticipatory responses, embedded performance validity, inattentiveness, impulsivity, sustained attention, and vigilance [247,248]. It can successfully diagnose ADHD subtypes, including inattention, impulsivity, attentional failure due to depression or psychomotor retardation, and inconsistency [248,249]. TOVA is currently the best attention test because it is FDA approved, economical at ~USD 15, has greater than 99% compliance, and only takes ~22 min to complete [249]. In addition, TOVA does not have a significant practice effect, is easy to supplement with Conners’ checklist, and allows for diagnostic heterogeneity [247,249,250].

## 8. Neuropsychiatric/Global Dopamine Dysfunction

Virtually all SUD patients have some form of neuropsychiatric/global dopamine dysfunction in life. Premorbid neuropsychiatric medical findings that predict progression to SUD are not widely screened for or even known (Table 2). The range of neuropsychiatric consequences of SUD are extremely diverse and cannot be accurately or reliably measured without standardization of testing (Table 5). This results in the absence of early identification of high-risk individuals across primary care and the various specialties. If a BHC were a mandated part of neuropsychiatric medicine, then the premorbid states would be characterized. For example, cardiovascular disease has been greatly controlled by testing premorbid patients with no heart disease or only early disease (hypertension or hyperlipidemia), i.e., EKG, labs, and echocardiogram. This approach engages the patient in their treatment and has been repeated in diabetes (hemoglobin A1c), obesity (body mass index, bioimpedance), breast cancer (mammogram/ultrasound), cervical cancer (pap smear), asthma (pulmonary function tests), and routine blood work for anemia, kidney, liver disease, etc. In addition, the BHC would help destigmatize SUD brain disorders, which is a key step towards medical parity [30,31]. The individual brain function tests could also be called BFTs to parallel liver function tests (LFTs), thyroid function tests (TFTs), pulmonary function tests (PFTs), and kidney function tests (KFTs), etc. [30,31].

The Millon Clinical Multiaxial Inventory-III (MCMI-III) is a diagnostic tool that can quickly and efficiently screen patients for neuropsychiatric disorders (Table 6). The MCMI-III provides a measure of 24 Axis I and II disorders and clinical syndromes for adults undergoing psychological or psychiatric assessment/treatment [252,253]. It has 175 questions and 27 scales with 99% compliance [252,253]. Assessed MCMI 1–4 correlates with the DSM [252,253,254,255]. MCMI-III contains a lifetime interpretation guide that captures many past and future predictions of behavior [256]. Non-psychiatrists should ideally use MCMI-III because DSM 5 has over 500 diagnoses/severity, which makes it difficult for non-psychiatrists to use. Additionally, psychiatrists’ use of the DSM typically results in one to two diagnoses for SUD patients, which is often inaccurate and misleading because SUD patients are typically worse than any one or two diagnoses alone. The most common finding of the MCMI-III test is the rate of depression, ~10–20% [257]. We studied the impact of SUD on brain electrophysiology and found that SUD significantly worsens normal temporal lobe abnormalities [257]. The bitemporal injury of SUD increases mood disorders [257]. Our MCMI-III data have been shown to have a high predictive value of suicide rates and secondary mood and psychiatric disturbances [257]. Conventional data suggest that at least 25% of adolescents have a psychiatric disorder. This by far underestimates the problem, which is that adolescents have closer to 50% Axis 1 diagnoses and ~90% have an Axis 2 trait. The continued dumping of SUD patients to psychiatrists demonstrates the current health care system’s failure to coordinate brain health and medical comorbidities [36].

## 9. Neurological Imaging

SUD should be recognized as a brain disorder, and thus, neurological imaging should be implemented in its diagnosis and treatment. It is known that imaging is a very useful tool to help understand neurotransmitter interaction at regions of interest (ROI) in the brain, and over the past 40 years, electrophysiology has become the standard and most cost-effective method (Table 7) [258,259,260,261,262,263,264]. In fact, of all imaging methods, decreased p300 amplitude/latency, increased theta wave on qEEG, and abnormal evoked potential on visual and auditory processing are most established with electrophysiology (Table 7) [257,264,265,266,267,268,269,270,271,272]. Therefore, in terms of imaging, Quantitative Electroencephalogram (qEEG)/P300/Evoked Potential (EP) should become the standard in the diagnosis/treatment of SUD patients.

**Figure 1:**
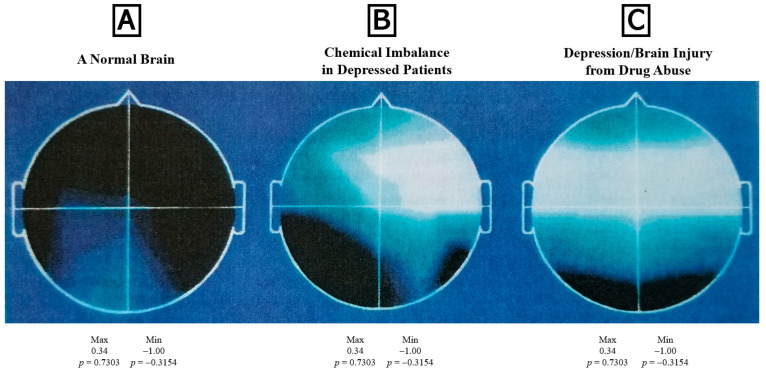
Clinical electroencephalography [257]. (**A**): Control/baseline. (**B**): Right frontal-temporal abnormalities typical of individuals with mood swings, cognitive decline, anxiety, and depression without SUD. (**C**): Significant SUD with worsening bitemporal damage [247,257,266,288,289,290,291]. (with permission from publisher).

Long-term successful treatment in medicine is accomplished with precision biological markers [257,278,279]. When all practitioners use the same test, e.g., EKG, echocardiogram, and cholesterol, etc., we obtain the best results [280,281,282]. The most common neuropsychiatric diagnosis in the US is depression, which only worsens with SUD [257,265,266]. Our laboratory showed that patients with both depression and SUD synergistically induce an exacerbated infraction in P300 metrics (Figure 1). Since SUD is a neurological disorder, talk therapy is an adjunct to spiritual and rehab therapeutics [182,259,260,261,262,263,264,283,284,285,286,287].

## 10. Changing the Metric Associated with SUD: Are We Going to the Promised Land

Addiction is a substantial health issue with limited treatment options approved by the FDA and, as such, currently available. It is known that cognitive circuitry and reward circuity overlap in the brain and share similar “light switches” [292]. The advent of neuroimaging techniques that link neurochemical and neurogenetic mechanisms to the reward circuitry brain function provides a framework for potential genomic-based therapies [293].

A search of the current literature (9-26-21) via PUBMED provides descriptions of promising new targets, alternatives such as animal models of gene therapy, addiction treatment and relapse prevention, and nutrigenomic and pharmacogenomic methods to manipulate transcription and gene expression [294]. In our opinion, while developing a cost-effective methodology to help primary care physicians incorporate the proposed BHC, we recognize the clinical benefit of early genetic testing to determine addiction risk stratification and dopaminergic agonistic (in subjects with hypodopaminergia) and antagonistic (in subjects with hyperdopaminergia) therapies. Adoption of these actionable targets, especially in the promotion of precision medicine, is potentially the genome-based wave of the future. In addition, further development, especially in gene transfer work (gene editing) and viral vector identification, could be the future of gene therapy for RDS. This could be accomplished through candidate and genome-wide association studies. Many gene clusters and polymorphisms implicated in drug, food, and behavioral dependence are linked by the common rubric RDS [293,294].

## 11. Risk Assessment Instruments to Identify Cognitive Brain Dysfunction

Since SUD is widely correlated with neuropsychiatric, medical, and social consequences, methods are reviewed to identify comorbidities and their high risk (Table 1, Table 2, Table 4 and Table 5) [36]. One very critical area of assessment must include a detailed family history of familiar SUD. The premorbid risk factors include virtually any neuropsychiatric/sleep disorders, traumatic brain injury, concussion, head trauma, family history of addiction, neurotransmitter and second messenger polymorphisms, hormonal abnormalities, and other areas of dysfunction (Table 2). Early identification of genetic risk for all reward dysregulation, either deficit or surfeit in terms of dopaminergic activity, is critical prior to any initiation of unwanted substances or even non-substance seeking as a preventive step [295].

Dopamine genotyping is critical for pediatrics because “an increase in dopamine levels is typical of all addictive drugs (final common SUD pathway) and sufficient to trigger forms of synaptic plasticity underlying adaptive behaviors” [296,297,298]. Genotyping predicts a higher probability of SUD in pediatric patients, but SUD is not completely determined by genes at any age [196,294,295,296]. Genes are a risk factor, particularly when young, but as individuals age, they transcend genetics with epigenetics. Therefore, the BHC is more essential long term.

## 12. Gateway to Reward Dysregulation: Reward Gene Antecedents to Abnormal Cravings

Drug and alcohol experimentation is almost endless, from bath salts to psychedelics (Table 8) [299,300,301,302,303]. Currently, despite the existence of the GARS test and possibly other viable genetically based panels, most clinicians involved in addiction medicine fail to identify SUD appropriately and objectively in patients of all ages. This conundrum translates to lack of psychometrics for evaluating the premorbid risks of SUD as part of primary care standard medicine [180].

Subjective medical histories of SUD and even medical patients alone are unreliable; for example, patients misreport their current height by 0.5–4 inches [333,334,335,336,337,338]. Testing of reward dysregulation such as SUD is marked by underreporting of patients not willing to testify against themselves, and moreover, there is difficulty in identifying the number of substances being used through standard testing, e.g., urine drug screens, breathalyzers, blood alcohol levels, etc. It is known that, for example, patients that carry the DRD2 A1 allele are more likely to deny or lie about their SUD issue [338]. Specifically, Comings et al. investigated the dopamine D2 receptor (DRD2) gene haplotypes, identified by the allele-specific polymerase chain reaction of two mutations (G/T and C/T) 241 base pairs apart, in 57 of the ATU subjects and 42 of the controls [338]. They reported that subjects with the one haplotype tended to show a decrease in mature and an increase in neurotic and immature defense styles compared to those without the one haplotype. These results suggest that the DRD2 locus is one factor controlling defense styles (lying).

The pediatric population will also benefit from a BHC. Currently, there is inadequate subjective and virtually no objective psychometrics to analyze the premorbid risks of SUD in the pediatric population (Table 3). However, according to NCDAS, ~50% of teenagers have experimented with illicit drugs at least once [12]. Early drug use/abuse has been shown to correlate with SUD later in life, and according to the gateway hypothesis, this early experimentation can escalate into more addictive illicit drugs later in adulthood [12,300,301,339]. Each episode of experimentation likely injures the brain to some degree, and eventually, these injuries accumulate, resulting in SUD [299,300,301,340]. Of interest in terms of a gateway hypothesis to SUD, Kandel and Kandel [339] describe how marijuana and other illicit drug use are preceded by tobacco or alcohol use. Additionally, using their mouse model, they were able to identify biological processes, showing that nicotine is a gateway drug that exerts a priming effect on cocaine through epigenetically increased global acetylation in the striatum [339].

It is noteworthy that it may be difficult in some cases to misdiagnose SUD with other conditions, and even careful following of DSM-5 may not provide the best possible manner to determine SUD compared with other neurological diseases. However, with the coupling of a strong family history and genetic addiction risk assessment, this might be a way to prevent misdiagnosis. Nevertheless, if we could agree that SUD is just a subset of a more major umbrella terminology such as reward deficiency, then it may not be such a real issue due to overall common reward circuitry dysregulation in general.

## 13. Conclusions

Once a person is in rehab or detox, it is the equivalent of their first stroke or heart attack, and by then, treatment is too late. The tools to prevent the progression of SUD are available and must be utilized; otherwise, the deaths attributed to addiction will continue to rise. The approach to brain health and addiction needs to be reexamined, and a new approach must be implemented by the medical community as a whole. At least three decades of clinical research reviewed here establishes a “Brain Health Check” based on precision medicine for the SUD phenotype centered around precision neuropsychiatric testing, i.e., Memory (CNSVS), Attention (TOVA), Neuropsychiatric (MCMI-III), Neurological Imaging (qEEG/P300/EP). The BHC establishes a set of objective assessments for brain health that parallel those used to treat cardiac disease and provides a form of standardization that is desperately needed. This approach would greatly improve the brain/mind health of all and help stop/prevent the rise of SUD with early detection and treatment, thus bringing an attenuation to the current addiction pandemic.

Moreover, The Carter Center has estimated that if the addiction crisis continues at the same rate, by the year 2030, it will cost America approximately 16 trillion dollars. The current status of our precious youth has been compromised by not only the COVID pandemic but also by the unfortunate state of an unwanted global opioid crisis, especially in America. Hundreds of thousands of people have succumbed to deadly opioid type drugs, a rate that is increasing yearly. The neurodevelopment of our children has been compromised by not only the victimization of mothers using opioids and other drugs during pregnancy but also by a high rate of DNA polymorphic antecedents as well as methylation on specific important genes related to normal brain function via known epigenetic insults. Along with these genetic antecedent insults affecting normal mRNA transcription and loss of required proteins to induce normal brain function, normal brain development in our youth is very complex.

It is generally accepted that myelination in the frontal cortex is quite delayed in our youth, especially as it relates to executive function and decision making. However, we embrace positive thinking, especially as it relates to neurotheology. An understanding of this short circuiting in brain development, along with potential high antecedent polymorphic risk variants or alleles and generational epigenetics, provides a clear rationale to embrace the Brain Research Commission (BRC) suggestion to mimic fitness programs with an adaptable Brain Health Check-up. This can be implemented in America and other countries’ educational systems. Adoption of this proposal may reduce juvenile criminal activities and potential attenuation of relapse to RDS behaviors.

## Figures and Tables

**Table 1:** Common medical comorbidities of SUD. ^1^

**Neurologic**	Dementia, Wernicke–Korsakoff Syndrome, Seizures, Cerebrovascular Accident, Cerebrovascular Disease, Polyneuropathy, Encephalopathy, Hepatic Encephalopathy, Head trauma, Sleep Disorders, Multiple Sclerosis, Neurodegenerative disorders, Neonatal Abstinence Syndrome
**Psychiatric**	Anxiety, Depression, Bipolar, Post Traumatic Stress Disorder, Attention Deficit Hyperactivity Disorder, Attention Deficit Disorder, Psychosis, Personality Disorders, Chronic Pain, Suicide, Sleep Disorder (decreased duration, REM, and CSF brain wash)
**Cardiovascular**	Hypertension, Coronary Atherosclerosis, Arrhythmias, Cardiomyopathy, Ischemic Heart Disease, Congestive Heart Failure, Myocardial infarction, Peripheral Vascular Disease
**Pulmonary**	Pneumonia, Aspiration Pneumonia, Asthma, Allergic Rhinitis, Toxic Rhinitis, Chronic Obstructive Pulmonary Disease, Lung Cancer
**Gastrointestinal**	Esophagitis, Mallory–Weiss Syndrome, Boerhaave Syndrome, Gastritis, Peptic Ulcer, Gallbladder Disease, Pancreatitis, Cirrhosis, Alcoholic Liver Disease, Nonalcoholic Fatty Liver Disease, Mild/moderate, Severe Liver Disease, Portal Hypertension, Intestinal Ischemia, Gastrointestinal Perforation, Inflammatory Bowel Disease
**Endocrine**	Diabetes, Obesity, Metabolic Syndrome, Hypothyroidism, Neuroendocrine abnormalities
**Infectious**	Hepatitis, Endocarditis, Bacterial Pneumonia, Tuberculosis, Skin Infections, Sexually Transmitted Diseases, Human Immunodeficiency Virus, Acquired Immunodeficiency Syndrome, Arthritis
**Hematologic**	Anemia, White Blood Cell Disorders, Platelet Disorders, Splenomegaly, Hyposplenism, Coagulopathy
**Nephrology**	Renal disease, Renal Failure, Fluid and Electrolyte Disorders
**Genitourinary**	Urinary Retention, Erectile Dysfunction
**Musculoskeletal**	Osteoporosis/penia, Sarcoporosis/penia, Fragility, Fibromyalgia, Temporomandibular Joint and Muscle disorders, Paralysis
**Dermatologic**	Scabies, Jaundice, Pruritus, Urticaria, Hyperpigmentation, Spider Telangiectasias, Angiomas, Caput Medusas. Flushing, Palmar Erythema, Psoriasis, Porphyria Cutanea Tarda, Leukonychia, Rhytids, Drug Injection Lesions (track marks)
**Social**	Trauma, Violent Behaviors, Criminal Behavior, Prison, Divorce, Homelessness, Internet Gaming
**Other**	Scleral Icterus, Cancer, Weight Loss, Inflammation
Ref: [51,54,55,56,57,58,59,60,61,62,63,64,65,66,67,68,69,70,71,72,73,74,75,76,77,78,79,80,81,82,83,84,85,86,87,88,89,90,91,92,93,94,95,96,97,98,99,100,101,102,103,104,105,106,107,108,109,110,111,112,113,114,115,116,117,118,119,120,121,122,123,124,125,126,127,128,129,130,131,132,133,134,135,136,137,138,139,140,141,142,143,144,145,146,147,148,149,150,151,152,153,154,155,156,157,158,159,160,161,162,163,164,165,166,167]	

^1^ Charlson–Deyo and Elixhauser-van Walraven (has SUD as a factor) Comorbidity Index scores consolidate the common medical comorbidities table (Table 1) into high-risk diagnoses, but if used alone miss 90% of secondary comorbidities caused by SUD [36,51]. REM: Rapid Eye Movement; CSF: Cerebral Spinal Fluid.

**Table 2:** SUD premorbid states.

**Cognitive**	ADHD, ADD, Impaired Memory, Impaired Judgment
**Neuropsychiatric Illness**	Any Psychiatric Illness, Head Concussion, Trauma, Chronic Traumatic Encephalopathy (CTE), Birth Injury, Sleep Disorders
**Social**	Low Education Attainment, Disturbed Family Life, Family History of Addiction, Abnormal Genetic Addiction Risk Scores, Amphetamine Use, Poverty, School Truancy, Unintended Pregnancy, Culture
REF: [32,33,34,53,169,170,171,172,173,174,175,176,177,178,179,180]	

**Table 3:** Parallel pattern to manage brain and cardiac disease. ^1^

Core Brain Domains/Tests	Core Cardiac Domains/Tests
Memory: CNSVS Attention: TOVA Neuropsychiatric: MCMI-III Genetic: GARS Imaging: Electrophysiology, qEEG/p300/EP	Blood pressure Blood work: cholesterol, CRP, etc. Electrophysiology: EKG Echocardiogram: Valves, Ejection fraction Imaging: CT angiogram
**Supplemental Brain Testing**	**Supplemental Cardiac Testing**
Memory: WMS, MCI Screening, MMSE Attention: Connors, ADD Checklist Neuropsychiatric: TT, MBTI, MMPI Imaging: MRI, PET, SPECT	Electrophysiological: Holter, EP testing Echocardiogram: Transesophageal Blood work: BNP, cardiac enzymes, etc. Imaging: Catheterization, MRI, PET

^1^ CNSVS: Central Nervous System Vital Signs, TOVA: Test of Variables of Attention, MCMI-III: Millon Clinical Multiaxial Inventory III, GARS: Genetic Addiction Risk Score, qEEG: Quantitative Electroencephalogram, EP: Evoked Potential, WMS: Wechsler Memory Scale, MCI: Mild Cognitive Impairment, MMSE: Mini-Mental State Examination, ADD: Attention Deficit Disorder, TT: Type and Temperament, MBTI: Myers–Briggs Type Indicator, MMPI: Minnesota Multiphasic Personality Inventory, MRI: Magnetic Resonance Imaging, PET: Positron Emission Tomography, SPECT: Single-Photon Emission Computed Tomography, CRP: C-Reactive Protein, BNP: B-type Natriuretic Peptide.

**Table 4:** Common areas of brain atrophy in SUD and MCI.

Amygdala	Midbrain
Anterior cingulate cortex	Nucleus Accumbens
Basal Ganglia	Occipital cortex
Cerebellum	Occipitoparietal cortex
Cingulate gyrus	Orbitofrontal cortex
Extended Amygdala	Parahippocampal gyrus
Frontal cingulate	Parietal cortex
Frontal Cortex	Prefrontal Cortex
Globus pallidus	Pulvinar
Insula	Putamen
Left temporal gyrus	Superior frontal gyrus
Medial Frontal Cortex	Thalamus
Mesencephalon	Ventral Tegmental Area
REF: [197,198,199,200,201,202,203,204,205,206,207,208,209,210,211,212,213,214,215,216,217,218,219,220,221,222,223,224,225,226]	

**Table 5:** Neuropsychiatric consequences of SUD. ^1^

Memory	Attention	Neuropsychiatric	IQ/Cognitive Considerations
↓Composite Memory ↓Verbal Memory ↓Visual Memory ↓Visual Immediate ↓Visual Delay ↓Auditory Immediate ↓Auditory Delay ↓Auditory Recognition Delay ↓General Memory ↓Working Memory ↓Spatial Memory ↓Declarative Memory ↓Visuoperception ↓Sensory Memory ↓Episodic Memory ↓Visuo-constructional abilities ↓Prospective Memory ↓Retro Memory	↓Attention ↓Complex Attention ↓Simple Visual Attention ↓Reaction Time ↓Response Time ↓Psychomotor Speed ↓Processing Speed ↓Motor Speed ↓Executive Function ↓Delayed Recall ↓Inattentiveness ↓Manual Dexterity ↓Inhibitory Control ↓Temporal Processing ↓Cognitive Flexibility ↑Impulsivity ↑Omission Errors ↑Commissions Errors Response Time Variability	Schizoid Avoidant Depressive Dependent Histrionic Narcissistic Antisocial Sadistic Compulsive Negativistic Masochistic Schizotypal Borderline Paranoid Anxiety Somatoform Bipolar Manic Dysthymia Alcohol Dependence Post-Traumatic Stress Thought Disorder Major Depression Delusional Disorder	↓IQ ↓Verbal IQ ↓Performance IQ ↓Abstract IQ ↓General Cognitive Functioning ↓General Intelligence ↓Reasoning
REF: [34,198,246,251,252]			

^1^ The range of neuropsychiatric consequences of SUD are extremely diverse and without uniformity of testing cannot be measured. The downward-facing arrows indicate decreases, and the upward-facing arrows indicate increases.

**Table 6:** Sample MCMI-III score report. ^1^

Category	BR Score	Diagnostic Scales
Modifying Indices	93	Disclosure
	20	Desirability
	90	Debasement
Clinical Personality Patterns	62	Schizoid
	83	Avoidant
	83	Depressive
	93	Dependent
	12	Histrionic
	40	Narcissistic
	66	Antisocial
	57	Sadistic
	16	Compulsive
	94	Negativistic
	65	Masochistic
Severe Personality Pathology	65	Schizotypal
	93	Borderline
	68	Paranoid
Clinical Syndromes	97	Anxiety
	66	Somatoform
	71	Bipolar: Manic
	88	Dysthymia
	68	Alcohol Dependence
	76	Drug Dependence
	76	Post-Traumatic Stress
Severe Clinical Syndromes	70	Thought disorder
	100	Major Depression
	63	Delusional Disorder

^1^ Significant scores are BR > 75 [256].

**Table 7:** Brain neuroimaging. ^1^

Brain Imaging Studies	Alterations in Brain Activation Patterns While Performing Cognitive Tasks
EEG/qEEG p300/EP (electrophysiology)	Delayed p300 latency Decreased voltage of p300 Abnormalities in auditory and visual evoked potentials Increased theta waves, decreased alpha and beta waves Abnormal polysomnography, i.e., increased nocturnal movement, decreased REM, decrease sleep efficiency
SPECT	Blood flow single-photon emission computed tomography Decreased prefrontal lobe and temporal lobe circulation Decreased cerebral circulation
fMRI	Abnormal diffuser tensor imaging Abnormal fiber connections Abnormal neuropsychological tasks Hypo-activation of neuronal networks Prefrontal, frontal, parietal regions
MRI	Smaller brain volume in 5 subcortical areas including amygdala, hippocampus, etc. SUD may have increased cortical thickness Brain Atrophy multiple regions (see Table 4) White Matter Microstructure
PET	Abnormal metabolism of dopamine and its transporters Abnormal binding to D2 receptors meg phase increases coherence beta gamma - Abnormalities in working memory - Anomalies in auditory and visual processing
REF: [198,251,257,264,265,266,267,268,269,270,271,272,273,274,275,276,277]	

^1^ Brain Health MAP imaging is very diverse, but over the past 40 years, electrophysiology has become the standard and the most cost-effective. Evoked potential = EP.

**Table 8:** The gateway of potential addictive substances and behaviors. ^1^

Illegal Substances	Legal Substances	Legal Foods	Abnormal Behavior
Synthetic Cannabinoid (Marijuana, CBD, Spice)	Synthetic Cannabinoid (Marijuana, CBD)	Caffeine	Gambling
Inhalants	Nicotine	Sugars	Internet
Anabolic Steroids	Alcohol	Carbohydrates	Sex
Ketamine	Anabolic Steroids	Salt	Shopping
Cocaine	Prescribed Opioids	Fats, Trans, Tallow	Thoughts/Feelings
Heroin	Benzodiazepine	Charcoaled food	Violence
Amphetamines (MDMA, Ecstasy)	Ketamine	Spiced foods	
Narcotics (Crocodil)	Narcotics	Canned Foods	
Psychedelics (LSD, Salvia, Mushrooms)	Barbiturates	Packaged Foods	
Synthetic Cathinones (Bath Salts, Flakka)	Sedative/hypnotics,	Processed Foods	
Miscellaneous (Kratom, Quaaludes, New Market Designer Drugs)	Miscellaneous (Kava, Kratom, Glue, Gasoline, etc.)		
REF: [2,13,173,304,305,306,307,308,309,310,311,312,313,314,315,316,317,318,319,320,321,322,323,324,325,326,327,328,329,330,331,332]			

^1^ Every drug has multiple methods of entry, e.g., vaping, snorting, combustible, and liquid, etc.
